# Exploitation of bacterial *N*-linked glycosylation to develop a novel recombinant glycoconjugate vaccine against *Francisella tularensis*

**DOI:** 10.1098/rsob.130002

**Published:** 2013-05

**Authors:** Jon Cuccui, Rebecca M. Thomas, Madeleine G. Moule, Riccardo V. D'Elia, Thomas R. Laws, Dominic C. Mills, Diane Williamson, Timothy P. Atkins, Joann L. Prior, Brendan W. Wren

**Affiliations:** 1 Department of Pathogen Molecular Biology, London School of Hygiene and Tropical Medicine, Keppel Street, London WC1E 7HT, UK; 2 Defence Science and Technology Laboratory, Porton Down, Salisbury, Wiltshire SP4 0JQ, UK; 3 School of Biosciences, University of Exeter, Devon, UK

**Keywords:** *Francisella tularensis*, vaccine, glycoconjugate, protein glycan coupling technology

## Abstract

Glycoconjugate-based vaccines have proved to be effective at producing long-lasting protection against numerous pathogens. Here, we describe the application of bacterial protein glycan coupling technology (PGCT) to generate a novel recombinant glycoconjugate vaccine*.* We demonstrate the conjugation of the *Francisella tularensis* O-antigen to the *Pseudomonas aeruginosa* carrier protein exotoxin A using the *Campylobacter jejuni* PglB oligosaccharyltransferase*.* The resultant recombinant *F. tularensis* glycoconjugate vaccine is expressed in *Escherichia coli* where yields of 3 mg l^−1^ of culture were routinely produced in a single-step purification process. Vaccination of BALB/c mice with the purified glycoconjugate boosted IgG levels and significantly increased the time to death upon subsequent challenge with *F. tularensis* subsp. *holarctica*. PGCT allows different polysaccharide and protein combinations to be produced recombinantly and could be easily applicable for the production of diverse glycoconjugate vaccines.

## 2. Introduction

Vaccines more than any other medical intervention measure have improved the lives of mankind. When selecting a vaccine, there are a number of choices. Live attenuated vaccines carry the advantage of exposing the host to an array of immunogenic epitopes, often leading to generation of humoral and cellular immunity; however, they also carry the risk of reversion to full virulence and they cannot be administered to immunosuppressed individuals. Killed vaccines are cheaper to store than live attenuated vaccines; however, only humoral immunity is induced resulting in the need to vaccinate individuals multiple times. A defining characteristic of a successful vaccine is the ability to evoke long-lasting protective immunity with minimal side effects. One strategy that has been particularly successful in achieving this goal is the use of glycoconjugate vaccines, which combine an immunogenic protein coupled to a glycan to induce both humoral and cellular immune responses. Additionally, subunit vaccines can be administered to immunosuppressed individuals. Examples of currently licensed human glycoconjugate vaccines include those against *Haemophilus influenzae, Neisseria meningitidis* and *Streptococcus pneumoniae,* in which bacterial polysaccharides are chemically bound to carrier proteins [1,2]. In the case of the *H. influenza* type B (Hib) vaccine, the carrier protein iCRM197 is a non-toxic version of diphtheria toxin isolated from *Corynebacterium diphtheriae* [3]. This same carrier protein is used in the Prevnar 13-valent capsule-based glycoconjugate vaccine that can be administered to infants as well as adults to protect against invasive disease caused by *S. pneumoniae* [4]. Although these glycoconjugate vaccines are extremely effective, their production using current technology requires both the purification of polysaccharide glycan from the native pathogen and the chemical coupling of the sugar to a suitable protein carrier, which can lead to impure products and batch variation between glycoconjugate preparations. In addition, the multistep process is expensive, time consuming and does not facilitate the construction and testing of diverse protein/glycan combinations. Here, we describe the development and optimization of a recombinant approach to the production of glycoconjugate vaccines that circumvents these problems to provide an inexhaustible supply of purified product.

**Figure 1.**
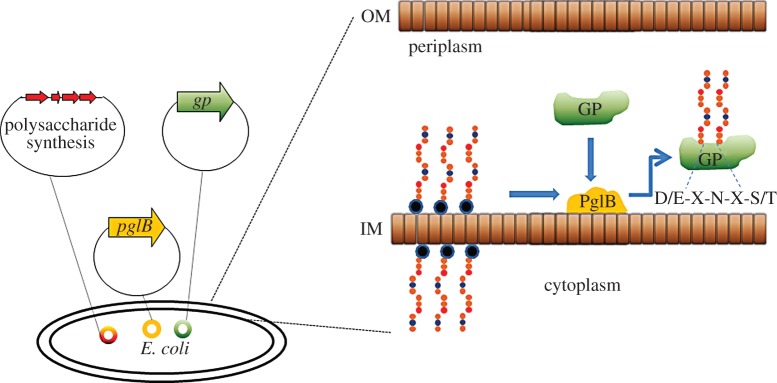
Principles of protein glycan coupling technology in *E. coli.* An *E. coli* cell is transformed with three plasmids to generate the cloned glycoconjugate protein (GP). The plasmids carry the oligosaccharyltransferase PglB, the biosynthetic polysaccharide locus and the carrier protein. The polysaccharide is synthesized on an undecaprenol pyrophosphate lipid anchor (blue/black circle) within the cytoplasm; this is transferred to the periplasmic compartment where PglB recognizes the lipid linked reducing end sugar and transfers the polysaccharide *en bloc* onto an acceptor sequon (D/E-X-N-X-S/T) on the carrier protein to produce the GP. IM, inner membrane; OM, outer membrane.

In recent years, general glycosylation systems have been identified in several bacterial species. For example, in *Campylobacter jejuni* a 16-kb gene locus that encodes a general *N*-linked glycosylation system has been characterized and found to modify over 50 proteins with a heptasaccharide glycan [5,6]. Remarkably, the general glycosylation locus functions in *Escherichia coli* and can be used to couple some glycans to a variety of proteins including non-*Campylobacter* proteins, as long as an appropriate acceptor sequon D/E-X-N-Y-S/T (where Y and X are any amino acid except P) is present on the acceptor protein [7,8]. The oligosaccharyltransferase (CjPglB) in the locus can transfer alternative glycans from other organisms, implying a relaxed specificity for the enzyme [9]. This relaxed reducing end sugar specificity has been demonstrated for *N*-acetylgalactosamine from the O-antigen of *E. coli* O157, *N*-aceytlglucosamine from the O-antigen of *E. coli* O7, O16 and O9a, *N*-acetylfucosamine from the O-antigen of *Pseudomonas aeruginosa* O11 and bacillosamine (2,4-diacetamido-2,4,6-trideoxyglucose) from the native *C. jejuni* heptasaccharide [9–11]. These developments have demonstrated the potential for producing novel combinations of glycoconjugates and have been termed bacterial protein glycan coupling technology (PGCT; figure 1) [12,13]. PGCT provides an efficient cloning method of glycoconjugate production in the widely used bacterium *E. coli.* Recent examples include the transfer of the *Yersinia enterocolitica* 09 O-antigen to the *C. jejuni* acceptor protein AcrA [14] and the *Shigella dysenteriae* O-antigen to a *P. aeruginosa* exotoxin A (ExoA) variant [15]. However, these novel protein/glycan combinations have not been reported to be protective and the potential for PGCT in glycoconjugate vaccine production has yet to be realized.

*Francisella tularensis* is a Gram-negative, facultative coccobacillus. It is the causative agent of tularaemia and is considered one of the most infectious bacteria known to man, yet there is currently no licensed vaccine available [16]. A single inhaled bacillus is capable of producing infection in 40–50% of individuals, with a mortality rate of up to 30 per cent [17]. There are two strains pathogenic to humans, *F. tularensis* subspecies *tularensis* or type A, which is associated with illness in North America, and *F. tularensis* subspecies *holarctica,* also known as type B, which is less virulent but has a higher global burden and is associated with infections in Europe [18]. *F. tularensis* is also classified as a class A bioterrorism agent based on its low infectious dose, high fatality rate and ease of aerosol distribution, making vaccine development a high priority for national security interests [19]. The most promising candidate to date has been a live vaccine strain (LVS) derived from subspecies *holarctica* that has been tested in human volunteers [20–22]. However, this vaccine is still capable of causing disease in murine infection models [23] and provides only partial protection against the most virulent type A strains [24]. Therefore, a vaccine capable of conferring protection against both types A and B of *F. tularensis* remains an imperative.

The protective efficiency of a glycoconjugate vaccine relies on the ability of a glycan to induce a strong immune response, and it has previously been shown that vaccination with purified lipopolysaccharide (LPS) from *F. tularensis* LVS afforded protection against subsequent *F. tularensis* LVS challenge in the murine tularaemia infection model [25]. The prevalent immune response was humoral [26], although recently *F. tularensis* LPS has been found to cause proliferation of a rare subset of B-1a lymphocytes cells in the absence of T-cell help or TLR4 stimulation in mice, indicating that the immune response to this antigen cannot be classified either as innate or adaptive [27]. The polysaccharide moiety alone is insufficient for long-lasting immunological memory cell proliferation [28], but several studies have confirmed that this organism's O-antigen repeat unit is an important immunogen [25,26,29,30] and it would be predicted that coupling the O-antigen to an immunogenic protein carrier would provide a longer lasting T-cell dependent immune response. However, the chemical conjugation method used and the handling of highly infectious agents makes the purification and production of these vaccines extremely difficult.

In this study, we clone and express the *F. tularensis* O-antigen coding region in *E. coli* and transfer it to the acceptor protein ExoA from *P. aeruginosa* using PGCT. We show for the first time that the *Francisella tularensis* O-antigen can be transferred by PGCT, and have produced a novel candidate vaccine in the murine *F. tularensis* infection model. The purified recombinant glycoconjugate vaccine was easily purified in high yields and was capable of providing significant protection against subsequent challenge with a virulent wild-type strain of *F. tularensis* subsp. *holarctica*, demonstrating a considerable improvement over the LPS unconjugated vaccine. This demonstrates that PGCT can be used to generate efficacious glycoconjugate vaccines, and the technique could be adapted to a variety of other pathogens.

## 3. Material and methods

### 3.1. Bacterial strains and plasmids

*Escherichia coli* strains were grown in Luria-Bertani (LB) broth at 37°C, with shaking. Antibiotics were used at the following concentrations: tetracycline 20 µg ml^−1^, ampicillin 100 µg ml^−1^, spectinomycin 80 µg ml^−1^ and chloramphenicol 30 µg ml^−1^. The host strain for initial cloning experiments was *E. coli* XL-1, subsequent strains used for glycoconjugate production were *E. coli* DH5α and CLM24 (see the electronic supplementary material, table S1). For efficacy studies, mice were challenged with *F. tularensis* subsp. *holarctica* strain HN63. *F. tularensis* HN63 was cultured on blood cysteine glucose agar (BCGA) plates (supplemented with 10 ml of 10% (w/v) histidine per litre) at 37°C for 18 h. The bacteria were suspended in phosphate-buffered saline (PBS) to an OD_550 nm_ of 0.1, equivalent to 1 × 10^9^ colony forming units (CFU) per ml. Dilutions were made in PBS and mice were infected with 100 CFU via the intra-peritoneal (IP) route.

### 3.2. Cloning, sequencing and expression of the *Francisella tularensis* O-antigen coding region

DNA was prepared from the *F. tularensis* subsp. *tularensis* strain SchuS4 by phenol extraction as described by Karlsson *et al*. [31]. The O-antigen coding region was amplified using the primers FTfragment2rev (5′-GGATCATTAATAGCTAAATGTAGTGCTG-3′) and Oant1ftfwd (5′-TTTTGAATTCTACAGGCTGTCAATGGAGAATG-3′) using the following cycling conditions: 94°C, 15 s, 55°C, 15 s, 68°C, 20 min; 35 cycles using Accuprime *Taq* Hifi (Invitrogen, UK). This was cloned into the TA cloning vector pGEM-T Easy to generate the vector pGAB1. The plasmid pGAB1 was digested with *Eco*RI in order to subclone the insert into the vector pLAFR to generate the construct pGAB2.

### 3.3. Immunofluorescence imaging of *Escherichia coli* cells carrying *Francisella tularensis* O-antigen-coding region

Approximately 1 × 10^9^ CFU of *E. coli* DH5*α* cells were fixed on separate glass cover slips by air-drying and washed in PBS. Cover slips were then incubated in 5% v/v fetal calf serum (FCS) in PBS for 2 h. After three 2 min washes in PBS, cover slips were incubated with IgG2a mouse monoclonal antibody (mAb) FB11 (1 μl ml^−1^ in 5% (v/v) FCS/PBS) for 1 h at 37°C and, following three washes in PBS, bacteria were air dried on a glass coverslip and probed with Alexa Fluor 488 goat anti-mouse IgG (whole molecule, Invitrogen Life Technologies Corp.). Cover slips were mounted on a glass slide using Vectashield mounting medium containing the DNA-specific counter stain 4′, 6-diamidino-2-phenylindole (DAPI). The level of fluorescence for each isolate was observed under fluorescent microscopy using an Olympus FluoView laser scanning microscope (Olympus Imaging and Audio Ltd).

### 3.4. Immunoblot analysis

To verify the transfer and presence of the *F. tularensis* O-antigen, samples were analysed by western blotting. *Escherichia coli* cells were grown overnight in 10 ml LB broth and subsequently diluted to an OD_600 nm_ of 1.0. Cells were isolated by centrifugation at 12 100*g* for 10 min, the supernatant was removed and cells were resuspended in 100 µl Laemmli buffer. Cells were lysed by boiling for 10 min before analysis by western blotting or silver staining. Mouse anti-*F. tularensis* O-antigen monoclonal antibody FB011 (AbCam, UK) was used at a dilution of 1 : 1000, and rabbit anti-HIS monoclonal antibody was used to detect ExoA at a dilution of 1 : 10 000. Secondary antibodies used were goat anti-mouse IRDye680 and IRDye800 conjugates used at 1 : 5000 dilutions. Signal detection was undertaken using the Odyssey LI-COR detection system (LI-COR Biosciences GmbH).

### 3.5. Determination of glycosylation sequon occupancy within exotoxin A

To establish if the asparagine residues at amino acid positions 262 and 404 within ExoA were both capable of being glycosylated with the *F. tularensis* O-antigen, these residues were altered to glutamine (_260_DNNNS_264_ altered to _260_DNQNS_264_ and _402_DQNRT_406_ altered to _402_DQQRT_406_) using an Invitrogen QuikChange XL kit according to manufacturer's instructions (Invitrogen Life Technologies Corp.).

The primers a826c_t828g 5′-gacctggacatcaaggataatcaga-3′ and a826c_t828g_ant 5′-atgaccgtgggagtagaattctgatt-3′ were used to mutate the _260_DNNNS_264_ to yield pGVXN150_260_ DNQNS_264_ and the primers a1252c_t1254g 5′-tgccccgtcgccaaagatcaacagagaactaaaggggaatg-3′ and a1252c_t1254g_a 5′-cattcccctttagttctctgttgatctttggcgacggggca-3′ to mutate _402_DQNRT_406_ to yield pGVXN150_402_DQQRT_406_. The construct carrying the glutamine modification at amino acid position 262 was subjected to a second round of QuikChange to generate pGVXN150_260_DNQNS_264_/_402_DQQRT_406_ to act as a negative glycosylation control. All modified constructs were generate using the following cycling conditions: one cycle of 95°C per 1 min, followed by 18 cycles of 95°C per 50 s, 60°C per 50 s, 68°C per 7 min 30 s and a final step of 68°C per 1 min.

*Escherichia coli* CLM24 carrying the vectors pGAB2, pGVXN114 and pGVXN150 or the glycosylation sequon modified pGVXN150_260_DNQNS_264_/pGVXN150_402_DQQRT_406_/pGVXN150_260_DNQNS_264_/_402_DQQRT_406_ was grown for 16 h in 10 ml LB broth at 37°C, with shaking. 0.5 ml of this culture was used to inoculate 9.5 ml of LB broth and further incubated with shaking at 37°C until an OD_600_ reading of 0.4–0.6 was reached. At this point, l-arabinose was added to a final concentration of 0.2 per cent and isopropyl β-d-1-thiogalactopyranoside (IPTG) to a final concentration of 1 mM to induce expression of *exoA* and *CjpglB,* respectively. After 4 h of induction, cells were pelleted by centrifugation at 12 100*g* for 10 min. Cells were resuspended in 1 ml 50 mM NaH_2_PO_4_, 300 mM NaCl, 10 mM imidazole, pH 8.0 supplemented with 0.1 per cent Tween, 1 mg ml^−1^ lysozyme and 1 µl ml^−1^ Benzonase nuclease (Novagen) and lysed using a Bioruptor sonicator (Diagenode, UK) on high settings on a cycle consisting of 30 s on and 30 s off for 15 min. The resulting lysate was centrifuged at 12 100*g* for 10 min and the supernatant transferred to a fresh 1.5 ml tube before 50 µl of Ni-NTA agarose (QIAGEN) was added. The agarose lysate mixture was incubated at 4°C for 1 h before being loaded into microcentrifuge spin cups (Thermo Scientific, Pierce Spin Cups, Thermo Scientific Inc.) and centrifuged at 1670*g* for 2 min. His-tagged ExoA was purified according to manufacturer's instructions (QIA expressionist, QIAGEN); protein was eluted in 50 µl of elution buffer containing 250 mM imidazole.

Purified protein was tested by western blotting as described above.

### 3.6. Production and purification of glycoconjugate vaccine

*Escherichia coli* CLM24 carrying the vectors pGAB2, pGVXN114 and pGVXN150 was grown as described above with some modifications. *Escherichia coli* cells were grown for 16 h in 200 ml LB broth at 37°C, with shaking. This was used to inoculate 1.8 l of LB broth and further incubated with shaking at 37°C until an OD_600_ reading of 0.4–0.6 was reached. At this point l-arabinose was added to a final concentration of 0.2 per cent and IPTG to a final concentration of 1 mM to induce expression of *exoA* and *CjpglB*, respectively; after another 5 h of incubation, 0.2 per cent l-arabinose was added again and the culture left to incubate overnight.

Cells were harvested by centrifugation at 5300*g* for 30 min, and pelleted cells were incubated at room temperature for 30 min in a lysis solution composed of 10× BugBuster protein extraction reagent (Novagen) diluted to 1× in 50 mM NaH_2_PO_4_, 300 mM NaCl, 10 mM imidazole, pH 8.0 supplemented with 0.1 per cent Tween, 1 mg ml^−1^ lysozyme and 1 µl ml^−1^ Benzonase nuclease (Novagen). Cell debris was removed by centrifugation at 7840*g* for 30 min, the supernatant was collected and 1 ml Ni-NTA agarose (QIAGEN) was added to the supernatant. The slurry-lysate was incubated for 1 h at 4°C with shaking then loaded into 10 ml polypropylene columns (Thermo Scientific). His-tagged ExoA was purified by the addition of an elution buffer according to manufacturer's instructions (QIA expressionist, QIAGEN) containing 250 mM imidazole with the addition of 20 per cent glycerol and 5 per cent glucose. Protein yields were estimated using a bicinchonic acid assay kit according to manufacturer's instructions (Pierce Biotechnology BCA Protein Assay Kit, USA).

For large-scale protein purification, material was isolated using GE Healthcare HIS trap columns and an AKTA purifier with an imidazole gradient of 30 to 500 mM. The collected fraction containing ExoA glycosylated with *F. tularensis* O-antigen was further purified using a resource Q anionic exchange column (GE Healthcare) with a NaCl gradient from 0 to 500 mM in 20 mM Tris HCl pH 8.0. This generated a typical yield of 2–3 mg ml^−1^ of glycoconjugate per 2 l of *E. coli* culture.

The same techniques were used for the generation of the ‘sham’ *C. jejuni* heptasaccharide ExoA glycoconjugate. The plasmid coding for this heptasaccharide was pACYCpgl carrying the entire *Cjpgl* cluster from *C. jejuni* 81116 [5].

### 3.7. BALB/c mouse challenge studies

Female Balb/c mice were obtained from Charles River Laboratories (Kent, UK) at six to eight weeks of age. The pilot study was done in groups of 10 mice immunized with either 0.5 µg *F. tularensis* LPS, 0.5 µg *F. tularensis* glycoconjugate, 0.5 µg *F. tularensis* glycoconjugate + Sigma Adjuvant System (SAS), 0.5 µg ‘sham’ glycoconjugate + SAS, 0.5 µg ‘sham’ glycoconjugate or SAS only. In addition, one group of mice were left untreated as challenge efficacy controls. The amount of SAS used was a 1 : 1 ratio (for example, 50 µl bioconjugate in PBS + 50 µl SAS).

The SAS solution was made by re-suspending 1 vial containing 0.5 mg monophosphoryl lipid A (detoxified endotoxin) from *Salmonella minnesota* and 0.5 mg synthetic trehalose dicorynomycolate in 2 per cent oil (squalene)–Tween 80–water in 1 ml PBS.

Immunizations occurred on days 0, 14 and 28 via the IP route. Mice were challenged 35 days after the last immunization with 100 CFU of *F. tularensis* strain HN63 by the IP route, delivered in 0.1 ml. Subsequent experiments used the same vaccination schedules except for increasing the number of mice per group to 15 and increasing doses to 10 µg of material per animal per immunization. Four weeks following final vaccination, five mice from each group were tail bled to obtain sera for antibody analysis. On day 3 post-infection, the same five mice from each group were culled, and spleens were harvested and analysed for bacterial load and cytokine response. For organ load determination, spleen samples were homogenized in 2 ml of PBS through 40 µm cell sieves (BD Biosciences). Cell suspension of 100 µl aliquots  were plated onto BCGA plates for the enumeration of bacteria. All work was performed under the regulations of the UK Home Office Scientific Procedures Act (1986).

### 3.8. Immunology

*Francisella tularensis* LPS-specific IgM and total IgG levels were determined in serum samples by ELISA as previously described [32]. Spleen supernatants were assessed using mouse inflammatory cytometric bead array kit for interleukin (IL)-10, IL-12p70, interferon-γ, IL-6, tumour necrosis factor-α, and monocyte chemoattractant protein-1 in accordance with manufacturer's instructions (CBA; BD biosciences). Cytokine concentrations were measured via quantification of PE fluorescence of samples in reference to a standard curve using a BD FACS Canto flow cytometer (BD biosciences).

### 3.9. Statistical analysis

All figures were generated using the program GraphPad Prism v. 5.0. Statistical analyses were performed using the program PASW (SPSS release 18.0). Survival data were analysed by pair-wise log rank test stratified by experiment. Cytokine and bacterial load data were transformed to the logarithm 10 and analysed using a univariate general linear model, using Bonferroni's post-tests to further clarify significant differences.

## 4. Results

### 4.1. Expression of the *Francisella tularensis* SchuS4 O-antigen in *Escherichia coli* DH5α cells

**Figure 2.**
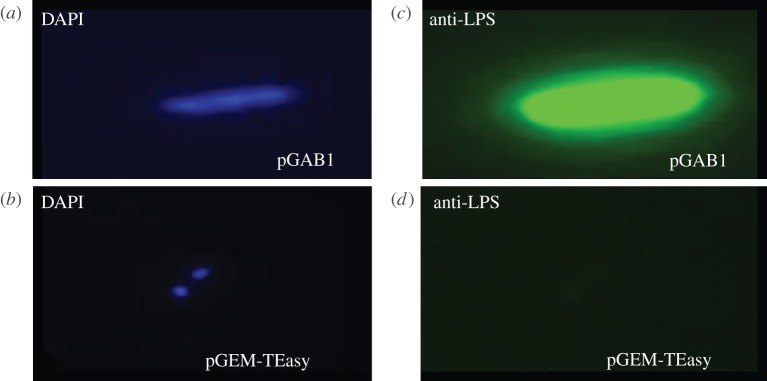
*Francisella tularensis* SchuS4 O-antigen is expressed in *E. coli* DH5α cells. *E. coli* cells carrying the *F. tularensis* vector pGAB1 (containing O-antigen coding region) and empty pGEM-T Easy vector, respectively, stained with DAPI to visualize nucleic acid in blue (*a*,*b*) and probed with mAb FB11 and Alexa Fluor 488 conjugated secondary antibody (*c*,*d*). Images are shown at 100× magnification.

The bacterial strains and vectors used in this study are summarized in the electronic supplementary material, table S1. The 20 kb *F. tularensis* SchuS4 O-antigen coding region was PCR amplified and cloned into pGEM-T Easy to generate the plasmid pGAB1. To confirm O-antigen expression and transport to the outer cell surface of *E. coli,* pGAB1 was transformed into DH5α cells and probed by immunofluorescence using mAb FB11, specific to the *F. tularensis* O-antigen. Figure 2*c* demonstrates the expression of the O-antigen on the surface of *E. coli* DH5α cells, which is absent in the vector-alone control (figure 2*d*). The presence of *E. coli* DH5α cells was confirmed by staining the respective samples with DAPI to visualize nucleic acid (figure 2*a*,*b*).

### 4.2. Oligosaccharyltransferase can transfer *Francisella tularensis* O-antigen to the acceptor protein exotoxin A

**Figure 3.**
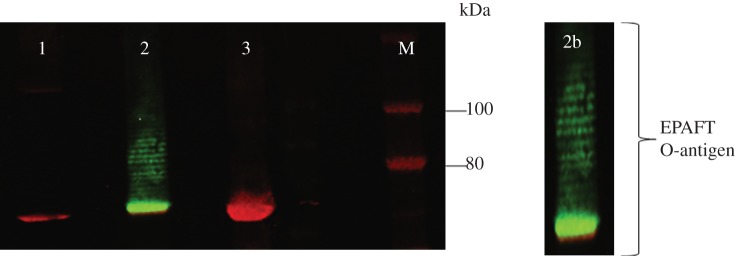
*F. tularensis* O-antigen is conjugated to ExoA by CjPglB in *E. coli* CLM24 cells. Two-colour immunoblots performed on His-tag purified ExoA using mouse mAb FB11 (green) and rabbit anti-6× His-tag antibody (red). Lane 1, *E. coli* CLM24 carrying pGAB2, pGVXN150, pGVXN115 (non-functional CjPglB control); lane 2, pGAB2, pGVXN150, pGVXN114 (functional CjPglB); lane 3, pGVXN150 only; panel 2b, close up view of His-tagged purified ExoA attached to various chain lengths of *F. tularensis* O-antigen. M = marker IRDye 680/800 protein marker. pGVXN150 contains ExoA, pGAB2 contains *F. tularensis* O-antigen, pGVXN114 and pGVXN115 carry a functional and non-functional CjPglB respectively.

In order to generate a strong IgG response and lasting immunity, a highly immunogenic protein is required as a carrier for the *F. tularensis* O-antigen. The selected carrier protein was an inactivated form of the ExoA variant (L552V, *Δ*E553) [15]. The protein was modified to carry two *N*-glycosylation sequons (N262 and N404), a C-terminal His tag and a signal peptide sequence from the *E. coli* protein DsbA fused to the N-terminal to localize ExoA to the periplasm of the *E. coli* host cell [15]. The *F. tularensis* O-antigen coding region was subcloned from pGAB1 into the unique *Eco*RI site of the low copy vector pLAFR1 [33] to yield the vector pGAB2. The plasmids pGAB2, pGVXN114 and pGVXN150 containing the O-antigen, CjPglB and ExoA, respectively, were transformed into *E. coli* strain CLM24 that has a deletion in the *waaL* gene rendering the *E. coli* host strain ligase negative and unable to transfer UndPP-linked glycan to lipid A, therefore generating a lipid-linked substrate specific for CjPglB. As negative glycosylation controls, CLM24 cells were transformed with pGVXN150 alone and also with the combination of pGAB2, pGVXN150 and pGVXN115, the latter coding for a version of CjPglB with point mutations within a domain involved in acceptor sequon recognition from _457_WWDYGY_462_ to _457_WAAYGY_462_ [5]. Following overnight induction of *CjpglB* and *exoA* expression with 1 mM IPTG and 0.2% l-arabinose (w/v), respectively, cells were lysed and His-tagged ExoA purified using a nickel column. Four elution fractions from each sample were separated by SDS PAGE and tested by immunoblotting using the mAb FB011 specific for *F. tularensis* LPS. A band matching the expected size of ExoA and an O-antigen-like pattern could only be purified when a functional CjPglB was present (figure 3, lanes 2 and 2b). In the absence of a functional CjPglB there was no cross-reaction with mAb FB11 (figure 3, lanes 1 and 3). Typical yields were 3 mg ml^−1^ of protein after the one step purification procedure. The His-tagged ExoA *F. tularensis* O-antigen conjugate (now termed glycoconjugate) was purified using Ni-NTA agarose (QIAGEN) and digested with Proteinase K. The disappearance of the O-antigen ladder after Proteinase K treatment but not in the untreated sample confirmed that the O-antigen was anchored to ExoA (see the electronic supplementary material, figure S1).

In order to test if *F. tularensis* O antigen was capable of glycosylating ExoA at both glycosylation sequons we generated site-directed mutants of the asparagine residues found at amino acid positions 262 and 404 and modified them to glutamines. A version of ExoA with both asparagines altered to glutamines was also generated as a negative control. The results from a 4 h induction of protein expression and small-scale protein purification demonstrated that both the _260_DNNNS_264_ and _402_DQNRT_406_ sequons can be occupied by the polysaccharide (see the electronic supplementary material, figure S2).

### 4.3. Vaccination with the glycoconjugate provides significant protection against *Francisella tularensis* subsp*. holarctica* infection in mice

**Figure 4.**
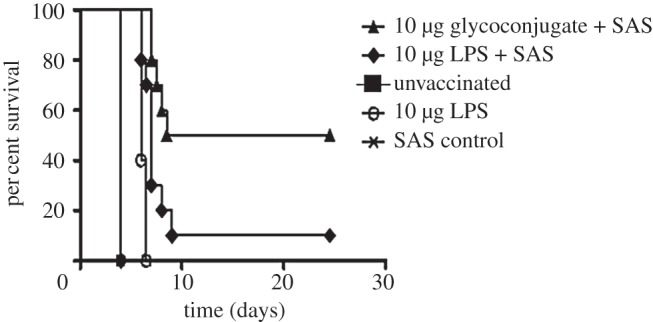
Vaccination with test glycoconjugate increases host survival compared with LPS and controls. Balb/C mice were vaccinated with three doses, two weeks apart, with glycoconjugate vaccine or relevant controls (*n* = 10 per group). Mice were challenged five weeks following final vaccination with 100 CFU of *F. tularensis* strain HN63 via the IP route. Mice were vaccinated with 10 µg of test glycoconjugate + SAS, LPS + SAS or 10 µg LPS and the data were analysed by stratified log rank test. Both LPS, LPS + SAS and test glycoconjugate provided improved protection when compared with the relevant unvaccinated controls (*p* < 0.001) and the SAS alone provided no survival benefit (*p* > 0.05). The test glycoconjugate provided significantly better protection than the LPS alone or LPS + SAS vaccine (*p* < 0.001 and *p* = 0.025, respectively).

In a pilot study we compared LPS alone against the glycoconjugate vaccine and monitored antibody levels and murine survival. In order to demonstrate the specificity of the glycoconjugate, we used controls that included mice with SAS adjuvant alone, unvaccinated naive mice and mice that were vaccinated with a ‘sham’ glycoconjugate control (*C. jejuni* heptasaccharide conjugated to ExoA). In this pilot study, the only vaccinated groups of mice that demonstrated increased survival compared with the appropriate controls were the 0.5 µg test glycoconjugate + SAS (*p* < 0.05) and 0.5 µg LPS (*p* < 0.001), determined by log rank test (see the electronic supplementary material, figure S3). These candidates were selected for further assessment at higher doses and a further group consisting of LPS + SAS was also added to determine if this combination matched the glycoconjugate + SAS group. Protection was compared between mice immunized with either 10 µg glycoconjugate + SAS, 10 µg LPS or 10 µg LPS + SAS. All three vaccines were protective when compared with the unvaccinated mice (*p* < 0.001), and the SAS adjuvant alone did not elicit any protection (*p* > 0.05; figure 4). This experiment also indicated that LPS + SAS did not elicit the same level of protection as the glycoconjugate + SAS combination (*p* < 0.05) and thereafter LPS + SAS was deemed unnecessary for testing. The study was repeated in order to provide further bacterial organ load and immunological response data.

### 4.4. Mice vaccinated with test glycoconjugate and challenged with *Francisella tularensis* subsp*. holarctica* have lower bacterial loads and pro-inflammatory cytokines 3 days post-challenge

**Figure 5.**
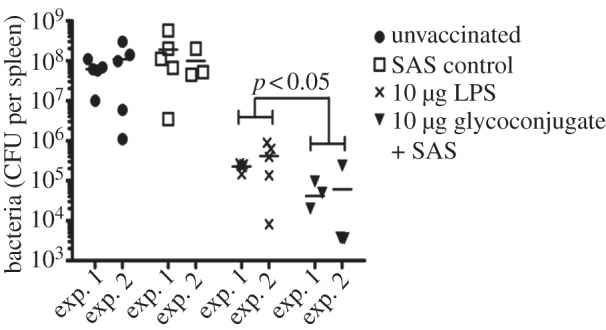
Mice vaccinated with test glycoconjugate show a reduced bacterial load in spleens compared with LPS and controls. Unvaccinated, SAS vaccinated, 10 µg LPS, or 10 µg test glycoconjugate + SAS vaccinated mice were challenged with 100 CFU of *F. tularensis* strain HN63 via the IP route. Spleens were removed 3 days post-infection from each group (*n* = 5) and assessed for bacterial CFUs. Logarithm data were analysed using a general linear model and Bonferroni's post-tests. There was no difference in bacterial load between SAS vaccinated and unvaccinated mice (*p* > 0.05) but the 10 µg LPS or 10 µg test glycoconjugate vaccinations had significantly decreased bacterial load when compared with relevant controls (*p* < 0.001). Mice vaccinated with the test glycoconjugate + SAS had significantly reduced bacterial numbers in the spleen compared with LPS (*p* < 0.05).

Three days post-challenge, five mice per group were sacrificed and bacterial loads in the spleens and the inflammatory responses were evaluated (figure 5). Figure 5 shows the bacterial loads from the spleens of mice vaccinated with 10 μg of each candidate. In both experiments, mice that were immunized with the glycoconjugate + SAS or LPS had significantly decreased bacterial loads in spleens (*p* < 0.01) when compared with the SAS and unvaccinated controls. In addition, when mice vaccinated with glycoconjugate + SAS were compared with those vaccinated with LPS alone, significantly less bacteria were enumerated in spleens (*p* < 0.05). Inflammatory cytokine profiles between the different vaccine groups were also analysed (see the electronic supplementary material, figure S4). Mice vaccinated with glycoconjugate + SAS and LPS alone had reduced levels of inflammatory cytokines when compared with the SAS and unvaccinated controls (*p* < 0.05), corresponding with the decreased bacterial loads. There was no significant difference between cytokine profiles for both experiments (*p* > 0.05).

### 4.5. Vaccination with the *Francisella tularensis* glycoconjugate induces a greater IgG immune response

**Figure 6.**
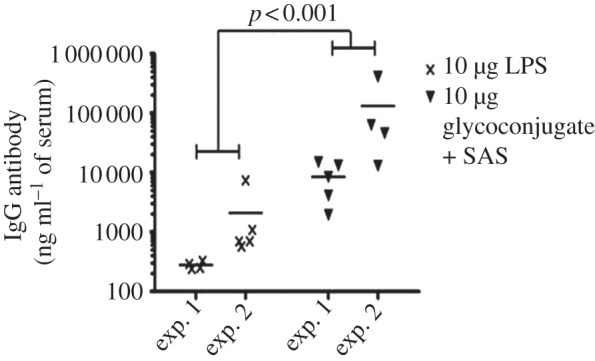
Increased IgG response in glycoconjugate vaccinated mice 7 days prior to challenge. Increased LPS-specific IgG was observed in the glycoconjugate + SAS vaccinated group when compared with mice vaccinated with LPS only (*p* < 0.001).

The levels of LPS-specific IgG were assessed in mice 7 days prior to challenge for both experiments. Increased LPS-specific IgG was observed in the glycoconjugate + SAS vaccinated group when compared with animals vaccinated with LPS only (*p* < 0.001). Although experiment 2 had higher levels of antibody (*p* < 0.01), we observed no evidence for the pattern between vaccination groups differing between experiments (*p* > 0.05; figure 6). No significant differences were observed between LPS-specific IgM levels from the glycoconjugate and LPS vaccine groups (see the electronic supplementary material, figure S5).

## 5. Discussion

Traditional glycoconjugate vaccine production using chemical conjugation requires that the glycan from the pathogenic organism is isolated and detoxified by stripping components such as lipid A, and that sufficient material is present to be chemically linked to a protein. The procedures involve harsh chemical treatments, can be extremely time consuming, often produce low yields, and are relatively expensive. In addition, the material generated at each step needs to be verified for purity and variation between vaccine batches is common. This process is also particularly difficult and hazardous for *F. tularensis* subsp. *tularensis* and *holarctica* owing to risks associated with aerosol generation and the low infectious dose. In this study, we used recombinant technology for the continuous generation of *F. tularensis* O-antigen within a safe laboratory strain of *E. coli* (figure 1).

The *F. tularensis* subsp. *tularensis* and subsp. *holarctica* repeating O-antigen subunit consists of a tetrasaccharide with the structure 4-α-d-GalNAcAN-(1–4)-α-d-GalNAcAN-(1–3)-β-d-QuiNAc-(1–2)-β-d-Qui4NFm-(1-), where GalNAcAN is 2-acetamido-2-deoxy-*O*-d-galact-uronamide, Qui4NFm is 4,6-dideoxy-4-formamido-d-glucose and the reducing end group QuiNAc is 2-acetamido-2,6-dideoxy-*O*-d-glucose [30]. Exploiting the relaxed sugar substrate specificity of CjPglB and the ability to target protein glycosylation, we generated a glycoconjugate vaccine by glycosylating ExoA from *P. aeruginosa* with the *F. tularensis* O-antigen repeat unit. This glycoconjugate vaccine was purified to a typical yield of 3 mg ml^−1^ of protein from a 2 l starting culture of *E. coli*. Thus, we demonstrate that the relaxed substrate specificity seen to be required by CjPglB extends to the ability to transfer glycans containing QuiNAc at their reducing ends.

PGCT has previously been used to produce a purified *S. dysenteriae* O-antigen/ExoA combination but the vaccine potential of this conjugate has not been reported [15]. More recently Iwashkiw *et al.* [14] used PGCT to link a *Y. enterocolitica* 09 polymer composed of *N*-formyl perosamine to a multidrug efflux pump component protein, AcrA, from *C. jejuni*. The construct was shown to be a useful diagnostic tool for *Brucella abortus* in bovine sera. The *F. tularensis* O-antigen/ExoA glycoconjugate produced in this study is the first report of a recombinantly generated glycoconjugate to be protective in an infectious disease model.

Vaccination with the test glycoconjugate was shown to provide protection against challenge with *F. tularensis* subsp. *holarctica* strain HN63, providing both an increased time to death compared with vaccination with LPS alone and significantly reduced bacterial loads (figures 4 and 5). The mice also had reduced inflammatory cytokine levels (see the electronic supplementary material, figure S4), correlating with the reduced bacterial load. This is indicative of an appropriate early host immune response to counteract the pathogen. Importantly, mice vaccinated with the glycoconjugate showed an altered immunological response compared with mice vaccinated with LPS alone. While the immune response against LPS is primarily a humoral immune response characterized by a dominance of IgM rather than IgG antibodies, the glycoconjugate + SAS vaccinated mice showed a large increase in the production of IgG (figure 6). Although increased IgG antibody level cannot be taken to mean direct evidence of a shift to a T-cell-dependent response, our results tentatively represent a proof of principle for the use of glycoconjugate vaccines against *F. tularensis* by demonstrating the expected shift in immune response from humoral to cell-mediated when the LPS O-antigen is conjugated to a protein and administered with an appropriate adjuvant.

The Sigma Adjuvant System was selected for use in this study because monophosphoryl lipid A (MPL)-based adjuvants have been demonstrated to be safe and efficacious immunostimulants in a number of vaccines including Cervarix (GlaxoSmithKline Ltd) [34]. The human papillomavirus vaccine Cervarix is licensed with an adjuvant combination of MPL and aluminium hydroxide. Furthermore, previous studies have shown that SAS is a suitable adjuvant for polysaccharide vaccine candidates for other intracellular pathogens [35].

Currently, the LVS-attenuated strain is the gold standard vaccine for *F. tularensis*. However, there are significant safety concerns before this vaccine can be licensed for use in humans. Sebastain *et al*. [36] attempted to incorporate the immunogenic components of the *F. tularensis* LVS vaccine whilst removing the adverse effects of the vaccine by combining an O-antigen–tetanus toxoid chemical conjugate with a highly attenuated *F. tularensis* LVS mutant that is unable to generate O-antigen. Unfortunately, while this vaccine improved the safety of the live vaccine, it conferred only partial protection in mice against intranasal challenge with wild-type strain SchuS4 [36]. Recently, Kim *et al*. [37] demonstrated that the *F. tularensis* LVS strain can be further attenuated by removing O-antigen polymerase function (Wzy) leading to a bacterium that is coated with a single repeat unit. This strain was capable of inducing high-level protection against type B *F. tularensis* in Balb/C mice following intranasal infection, but it did not match the LVS strain in terms of protection against type A *F. tularensis* challenge. However, the study demonstrates the importance of the O-antigen as an inducer of immunological response and the necessity to include this component in the vaccine, implying the necessity for a vaccine that induces both cellular and humoral immunity [37], such as a glycoconjugate.

The results from the present study represent a significant advance in the application of bacterial PGCT to produce successful glycoconjugate vaccines. The versatility and recombinant nature of this system will facilitate vaccine design. For example, additional glycosylation sites can be engineered into ExoA to improve the ratio of glycan to protein in the glycoconjugate. In these studies 10 μg of purified LPS were compared with 10 μg of total glycoconjugate measured by bicinchonic acid assay, an assay that detects only the concentration of protein and not of glycan. The mice vaccinated with glycoconjugate will have received substantially lower doses of O-antigen than the mice vaccinated with LPS. Increasing the amount of conjugated O-antigen given to mice could substantially improve efficacy. In addition our results demonstrate that both glycosylation sequons grafted within ExoA are capable of being occupied (see the electronic supplementary material, figure S2). The abundance of glycan signal seen near the size where unglycosylated ExoA normally migrates on an SDS PAGE gel shows that a significant amount of glycoprotein carries single repeat units. It is likely therefore that in this system, CjPglB competes with the *F. tularensis* O-antigen polymerase (Wzy). We are currently working on modulating the level of CjPglB to ensure that higher polymer length glycans are attached. An advantage of this technology over traditional chemical conjugation methods is that the protein to be glycosylated can easily be ‘swapped out’ and replaced with another protein of choice. This step requires only two protein modifications, consisting of a signal sequence to ensure it is delivered to the periplasm and the addition of a ‘glycosylation tag’ sequon at the N- or C- terminus. This means that several glycoconjugate combinations can be rapidly produced and purified and combined to provide multiple immunogenic epitopes within the vaccine. This is particularly significant as the intracellular pathogen *F. tularensis* has been notoriously difficult in deriving candidate antigens that protect against infection.

We are currently working towards modifying a number of immunogenic *F. tularensis* proteins to generate a dual-specific immune response against both the glycan and the protein. Future work will focus on using glycotags to optimize the efficacy of recombinant *F. tularensis* glycoconjugate vaccines. This study demonstrates the efficacy of a recombinant glycoconjugate vaccine produced by PGCT and provides a foundation for a new era in the generation of glycoconjugate vaccines.

## 6. Acknowledgements

We thank Dr Michael Wacker and Dr Michael Kowarik (GlycoVaxyn) for providing PGVXN114, pGVXN115, pGXVN150, pLAFR1 and invaluable suggestions. This work was supported by the Defence Science and Technology Laboratories and the Biotechnology and Biological Research Council, United Kingdom.

## Supplementary Material

Supplementary tables and figures
