# Supplementary material for: Exploitation of bacterial N-linked glycosylation to develop a novel recombinant glycoconjugate vaccine against Francisella tularensis
Source: Open Biol. 2013 May;3(5):130002. doi: 10.1098/rsob.130002 (PMC3866875; doi:10.1098/rsob.130002)
Supplement: Supplementary tables and figures [file rsob130002-s1.docx]

**SUPPLEMENTARY TABLES AND FIGURES:**

**Table S1.** Strains and plasmids used in this study

| Strain/plasmid | Description | Source |
| --- | --- | --- |
| *E. coli* DH5α | F-φ80*lac*ZΔM15 Δ(*lac*ZYA-*arg*F) U169 *deo*R*rec*A1 *end*A1 *hsd*R17 (rk-, mk+), *gal*- *pho*A*sup*E44λ – *thi*-1 *gyr*A96 *rel*A1 | Invitrogen |
| *E. coli* XL-1 | endA1 gyrA96(nalr)thi-1 relA1 lac gln V44 F’[::Tn10 proAB+ lacIq Δ (lacZ)M15] hsdR17 (r_k_^-^m_k_^+^) | Stratagene |
| *E. coli* CLM24 | rph-I IN(rrnD-rrnE) 1, ΔwaaL | [^9^](#_ENREF_9) |
| *F. tularensis* subs. *tularensis* strain SchuS4 | Type A strain | DSTL, Porton Down laboratories |
| *F. tularensis* subs. *holarctica* strain HN63 | Type B strain, isolated in Norway from an infected Hare | ^Green, M., et al., Efficacy of the live attenuated Francisella tularensis vaccine (LVS) in a murine model of disease. Vaccine, 2005. 23(20): p. 2680-6^ |
| pGEM-T Easy | TA cloning vector, amp^r^ | Promega |
| pLAFR1 | Low copy expression vector, tet*^r^* | [^31^](#_ENREF_31) |
| pGAB1 | *F. tularensis* O antigen coding region inserted into MCS of pGEM-T easy | This study |
| pGAB2 | *F. tularensis* subs. *tularensis* strain SchuS4 O antigen coding region inserted into EcorI site of pLAFR. | This study |
| pGVXN114 | Expression plasmid for C*j*PglB regulated from the Lac promoter in pEXT21. IPTG inducible, HA tag, Spec^r^. | GlycoVaxyn |
| pGVXN115 | Expression plasmid for *C. jejuni*non functionalPglB due to a mutation at _457_WWDYGY_462_ to _457_WAAYGY_462,_ regulated from the Lac promoter in pEXT21. IPTG inducible, HA tag, Spec^r^. | GlycoVaxyn |
| pGVXN150 | Expression plasmid for *Pseudomonas aeruginosa* PA103 (DSM111/) Exotoxin A with the signal peptide of the *E. coli*DsbA protein, two inserted bacterial N-glycosylation sites and a hexahis tag at the C-terminus. Induction under control of an arabinose inducible promoter, Amp^r^ | GlycoVaxyn |
| pGVXN150_260_DNQNS_264_ | Expression plasmid for *Pseudomonas aeruginosa* PA103 (DSM111/) Exotoxin A with the signal peptide of the *E. coli*DsbA protein, two inserted bacterial N-glycosylation sites, AA at position 262 altered from N to Q and a hexahis tag at the C-terminus. Induction under control of an arabinose inducible promoter, Amp^r^ | This study |
| pGVXN150_402_DQQRT_406_ | Expression plasmid for *Pseudomonas aeruginosa* PA103 (DSM111/) Exotoxin A with the signal peptide of the *E. coli*DsbA protein, two inserted bacterial N-glycosylation sites, AA at position 404 altered from N to Q and a hexahis tag at the C-terminus. Induction under control of an arabinose inducible promoter, Amp^r^ | This study |
| pGVXN150_260_DNQNS_264_/_402_DQQRT_406_ | Expression plasmid for *Pseudomonas aeruginosa* PA103 (DSM111/) Exotoxin A with the signal peptide of the *E. coli*DsbA protein, two inserted bacterial N-glycosylation sites, AA at position 262 and 404 altered from N to Q and a hexahis tag at the C-terminus. Induction under control of an arabinose inducible promoter, Amp^r^ | This study |
| pACYC*pgl* | pACYC184 carrying the *CjPglB* locus, Cm^r^ | [^5^](#_ENREF_5) |

**
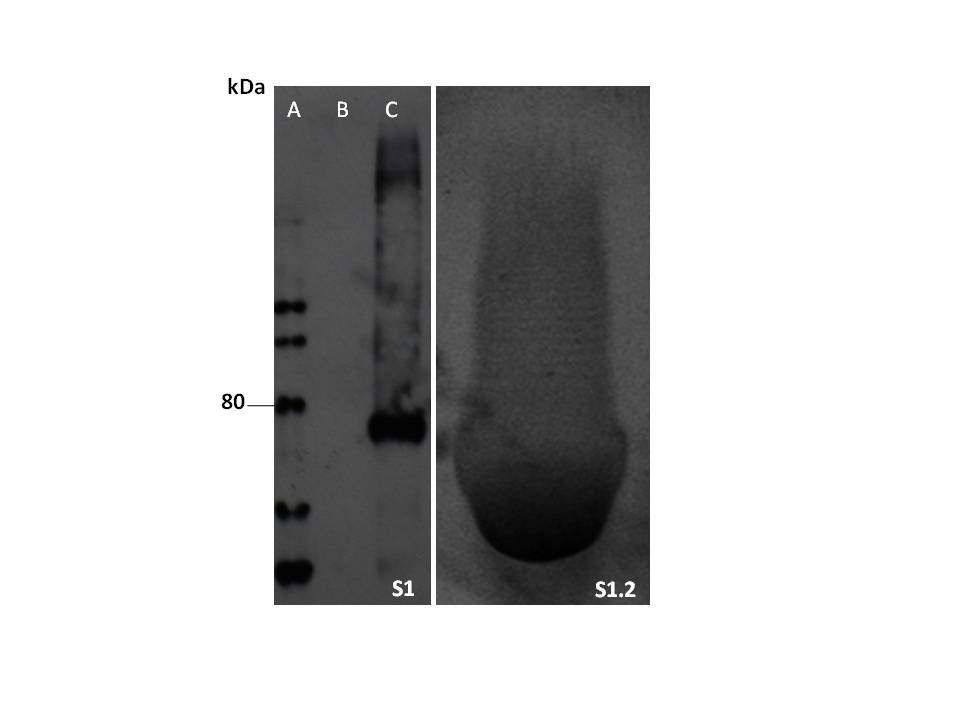
**

**Figure S1. The *F. tularensis* O-antigen is conjugated to ExoA.** Treatment of the glycoconjugate with proteinase K to degrade ExoA results in loss of the O-antigen ladder at the corresponding size. Western blot was performed with monoclonal antibody FB11. A, Markers; B, proteinase K digested ExoA *F. tularensis* O-antigen glycoconjugate; C, glycoconjugate heated to 50°C o/n without proteinase K. S1.2; Silver stained ExoA *F. tularensis* O-antigen glycoconjugate.


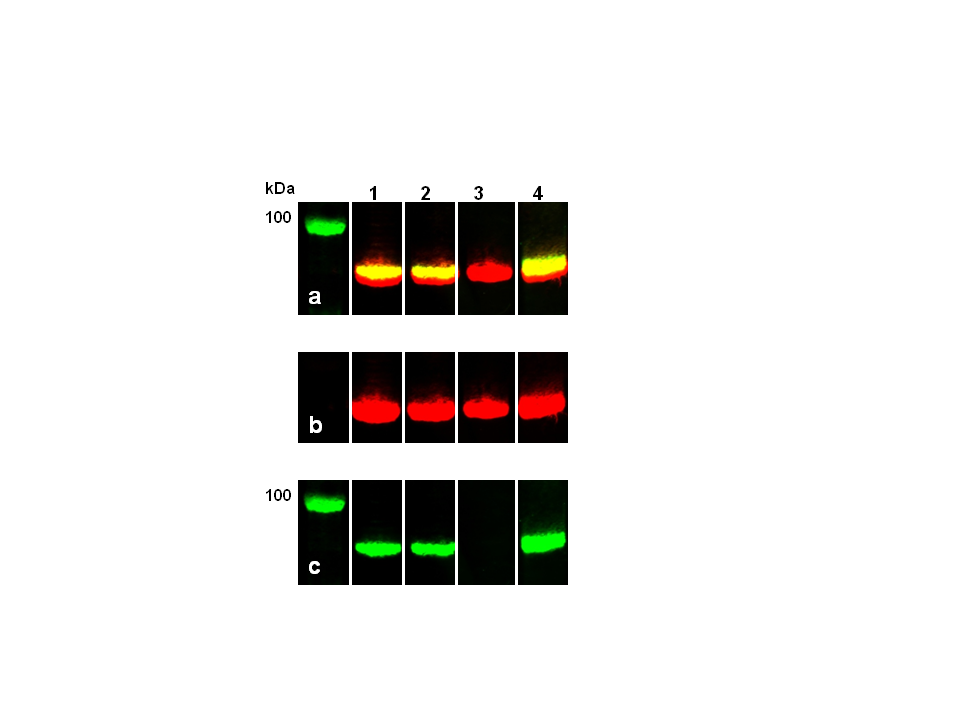


**Figure S2. Determination of glycosylation sequon occupancy within ExoA.** Panel a, combined anti glycan and anti HIS signal; panel b, anti HIS signal only; panel c, anti glycan signal only. Lane 1, _260_DNNNS_264_ altered to _260_DNQNS_264_; Lane 2, _402_DQNRT_406_ altered to _402_DQQRT_406_; Lane 3, _260_DNNNS_264_ altered to _260_DNQNS_264_ and _402_DQNRT_406_ altered to _402_DQQRT_406;_ Lane 4, exotoxin A encoded encoded from pGVXN150.


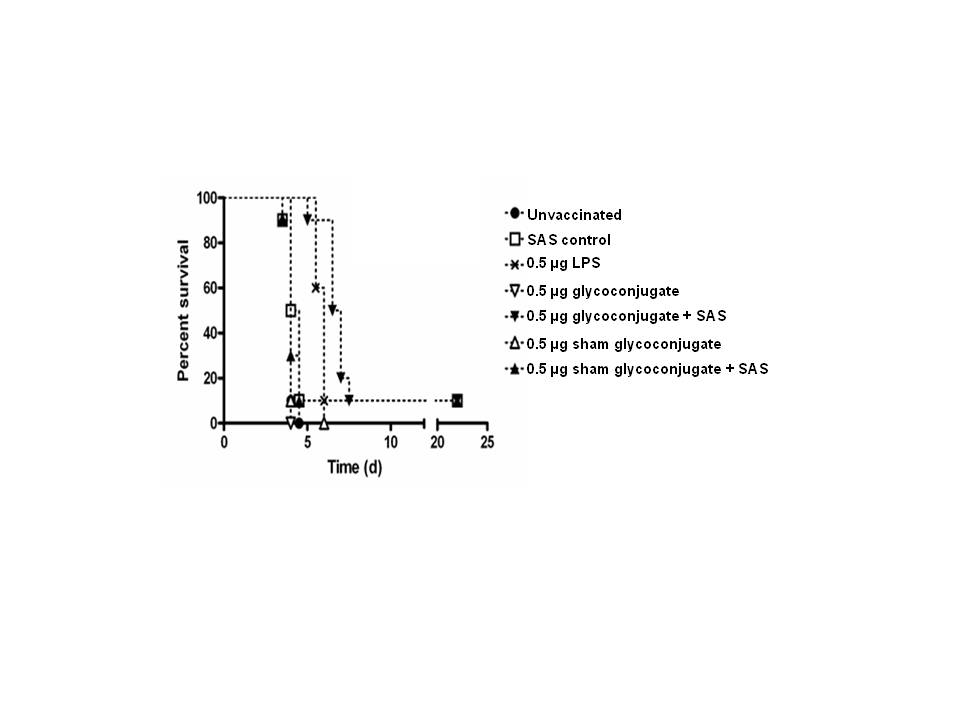


**Figure S3.** **Pilot study of vaccine candidates and relevant controls**. Balb/C mice were vaccinated with three doses, 2 weeks apart with candidate vaccine or relevant controls (n=10 per group). Mice were challenged 5 weeks following final vaccination with 100 CFU of *F. tularensis* strain HN63 via the i.p. route. 0.5 µg of product per time point were assessed. Mice vaccinated with 0.5 µg test glycoconjugate with SAS (P<0.05) and the 0.5 µg LPS vaccines (P<0.001) survived longer than controls as determined by log rank test. Glycoconjugate, *F. tularensis* O-antigen ExoA glycoconjugate; sham glycoconjugate, *C. jejuni* 81116 heptasaccharide ExoA glycoconjugate.


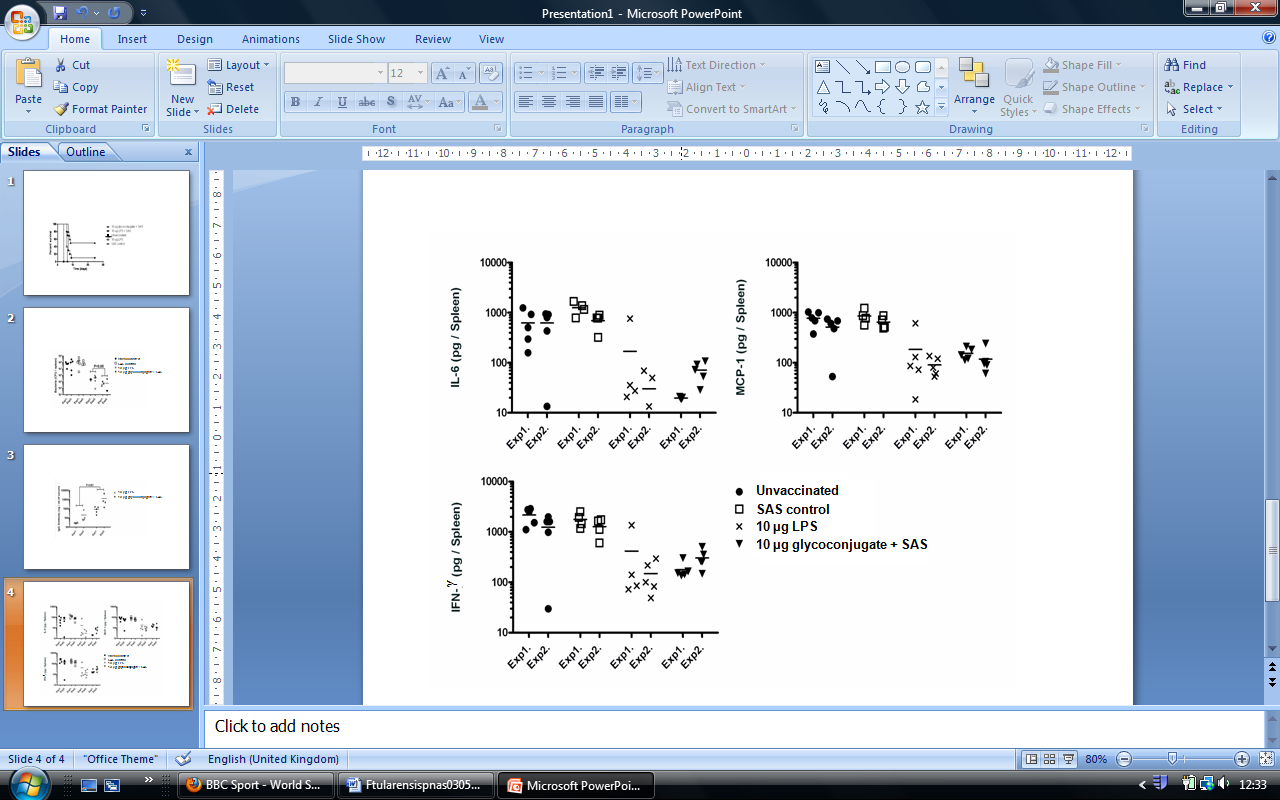


**Figure S4.** **Reduced inflammatory responses seen in LPS and glycoconjugate vaccinated mice compared to controls.**

Unvaccinated, SAS vaccinated, 10 µg LPS or 10 µg test glycoconjugate vaccinated mice were challenged with 100 CFU of *F. tularensis* strain HN63 via the i.p. route. Spleens were removed 3 days post infection from each group (n=5) and assessed for cytokine response. Levels of IL-6, MCP-1 and IFN-γ, were measures by CBA; all cytokine data pg/spleen. Individual points represent individual samples with line indicating the mean. Logarithm data was analysed using a general linear model and Bonferroni’s post tests. Cytokine production (IL-6, MCP-1 and IFN-γ) was comparable between controls (untreated and SAS) and the two vaccine treated groups (LPS and glycoconjugate). Cytokine concentration was reduced in vaccinated mice compared to relevant controls (P<0.05) and the experiments 1 and 2 did not differ from each other (P>0.05).


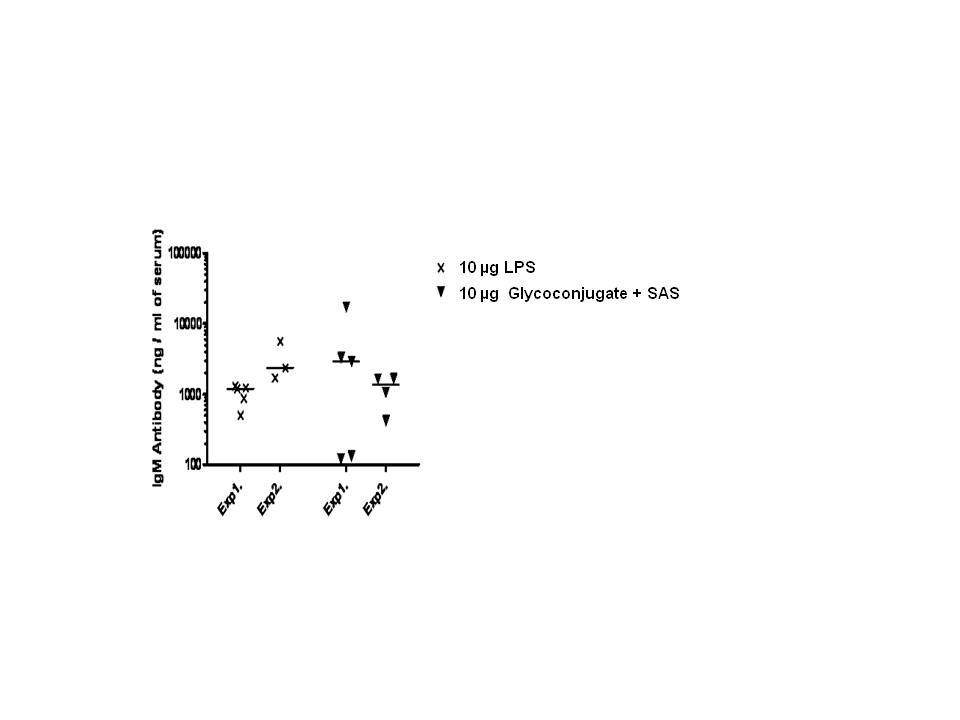


**Figure S5.** ***F. tularensis* LPS specific IgM levels observed in vaccinated mice 1 day prior to challenge.** There were no differences between LPS specific IgM levels in the glycoconjugate and SAS vaccinated group when compared to animals vaccinated with LPS only group (P>0.05).  We observed no evidence of the LPS-specific IgM titres differing between experiments (P>0.05).
